# A psychometric assessment of the St. George’s respiratory questionnaire in patients with COPD using rasch model analysis

**DOI:** 10.1186/s12955-015-0320-7

**Published:** 2015-08-20

**Authors:** Chyi Lo, Wen-Miin Liang, Liang-Wen Hang, Tai-Chin Wu, Yu-Jun Chang, Chih-Hung Chang

**Affiliations:** School of Nursing, China Medical University, Taichung, Taiwan R.O.C.; Graduate Institute of Biostatistics, China Medical University, Taichung, Taiwan R.O.C.; Department of Respiratory Therapy, College of Health Care, China Medical University, Taichung, Taiwan R.O.C.; Sleep Medicine Center, Department of Internal Medicine, China Medical University Hospital, Taichung, Taiwan R.O.C.; Department of Medical Affairs, Chang Gung Memorial Hospital at Kaohsiung, Kaohsiung, Taiwan R.O.C.; Epidemiology and Biostatistics Center, Changhua Christian Hospital, Changhua, Taiwan R.O.C.; Buehler Center on Aging, Health & Society, Feinberg School of Medicine, Northwestern University, 750 N. Lake Shore Drive, Suite 601, Chicago, IL 60611 USA; Department of Nursing, China Medical University Hospital, Taichung, Taiwan R.O.C.

## Abstract

**Background:**

The St. George’s Respiratory Questionnaire (SGRQ) was a widely used tool to assess disease impact on patients with obstructive airways disease. Although traditional methods have generally supported construct validity and internal consistency reliability of SGRQ, such methods cannot facilitate the evaluation of whether items are equivalent to different individuals. The purpose of this study is to rigorously examine the psychometric properties of the SGRQ in patients with chronic obstructive pulmonary disease (COPD) using Rasch model analysis.

**Methods:**

A methodological research was conducted on SGRQ in a sample of 240 male patients with COPD recruited from the outpatient services in Central Taiwan. The psychometric properties of the SGRQ were examined using Rasch model analysis with a mixed rating scale and partial credit mode by Winsteps software. The level of matching between the item’s difficulty and person’s ability was analyzed by item-person targeting as well as ceiling and floor effects. Item-person maps were also examined for checking the location of the item’s difficulty and person’s measures along the same scale. Finally, the differential item functioning (DIF) was examined to measure group equivalence associated with age and disease’s severity.

**Results:**

Each of the three domains (Symptom, Activity, Impact) of the SGRQ was found to be unidimensionality. The person separation index ranged from 1.21 (Symptom domain) to 2.50 (Activity domain). There was a good targeting for the SGRQ domains, except the Impact domain (1.36). The percentage of ceiling and floor effects were below 10 %, except the ceiling effect in the Impact domain (26.25 %). From item-person maps, gaps of location of item corresponded to patient’s ability were identified. The results have also showed that many items in SGRQ revealed age or severity related DIF.

**Conclusions:**

Except the Symptom domain of SGRQ, the others have a reliabile internal consistency and a good hierarchical structure. The results of Rasch model analysis can highlight aspects for scale improvement, such as gap, duplicate items or scale responses. There was some age or severity related DIF indicating somewhat unstable across different characteristics of group.

IRB No.: DMR94-IRB-179.

## Introduction

Chronic obstructive pulmonary disease (COPD) is one of the major causes of mortality worldwide and is associated with high level of disability [1]. COPD is a respiratory system disease with irreversible damage of pulmonary and bronchial tubes, represents the state of chronic airflow limitation [2]. It not only causes physiological discomfort but also has a psychosocial influence on individuals. The clinical assessment of COPD often involves measurement of lung function parameters (e.g., FEV1) and exacerbation level of a patient to evaluate the disease progress and the therapeutic effect [3]. However, the overall impact of COPD on individuals is multi-faceted and not entirely reflected by these clinical parameters. For this reason it is now realized that no single measure can adequately reflect the nature or severity of COPD and it often needs to be supplemented by other indicators from a patient’s perspective, such as those related to patient-report outcomes (PROs) or health-related quality of life (HRQOL). To date, evaluation of the treatment effect has emphasized the improvement of the quality of life rather than the small gains in survival rate or physiological indicators [4]. PROs have gradually become an important element and a crucial source for monitoring disease condition or assessing the effectiveness of treatment, especially in some health problems such as subjective discomfort and psychological distress [5]. Therefore, the U.S. Food and Drug Administration (FDA) has recommended that objective indicators combined with PROs be considered a more comprehensive form of outcome evaluation since 2006 [6]. However, most of the measurement of PRO relies primarily on the construction of a questionnaire. Clinicians and Researchers are quite concerned about how well a questionnaire was developed in order to accurately measure PRO with minimal error, thereby integrating it into clinical practice and increasing the quality of clinical service.

The St. George’s Respiratory Questionnaire (SGRQ) is one of a widely used PRO tool to assess disease impact on patients with obstructive airways diseases, such as asthma, COPD and bronchiectasis, and it has also been translated and adopted in many countries [7–9]. The SGRQ can provide a psychosocial impact profile of these patients that cannot be identified by the tests of lung function. Clinically, it has shown to be a valuable tool in quantifying the impact of chronic obstructive airways diseases on symptom, functional measures and well-being [10, 11] and in evaluating the effectiveness of health care [12].

Despite the demonstrated acceptable reliability and validity of the SGRQ, its data have been mostly validated using classical test theory (CTT) procedure. Although the CTT approach has been widely adopted in the psychological measurement, it also has some recognized shortcomings such as test or sample dependence [13]. That is, within CTT a person’s test score may easily vary depending on which test is being administered and, in turn, the difficulty of the same item depends on which sample is being assessed.

Nevertheless, modern test theory based models such as the Item Response Theory (IRT) can overcome these potential disadvantages. IRT, known as latent trait theory, utilizes probabilitistic model to construct a questionnaire based on the relationship between a person’s response to a question and his or her level on the construct (symbolized by θ) being measured by the scale. This relationship is conditional in that people with higher levels on the underlying construct will have a higher probability of endorsing response categories that are consistent with higher trait levels [13, 14]. Questionnaire constructed based on the IRT is superior to that of traditional CTT because IRT questionnaire is constructed using a model that take into consideration of both subject’s ability and degree of difficulty of test question. Therefore, the subject’s test score is not affected by the ability of the subject or difficulty of the test. i.e., the estimates of item location (difficulty) and person measures (ability) are independent regardless of respondents’ backgrounds or the items in a test [14].

Additionally, The difference between CTT and IRT is that CTT gives equal weight to all the items even though, in reality, there is different in the degree of difficulty. For instance, CTT gives the same one point to each of mountain climbing and walking on flat surface. Obviously, these two categories are quite difference in the degree of difficulty. The appropriateness of the total unweighted score as way to characterize a person is not taken for granted. On the other hand, IRT gives different point to each item depending on the difficulty of the question [14]. i.e., IRT allows the responses (raw scores) from different items representing different severity. Thus IRT model is that an individual’s response to any given item reveals a level of ability in the trait being measured.

Several studies have highlighted the advantages of Item Response Theory (IRT) over Classical Test Theory (CTT) methods [15, 16]. Rasch model is one of the family of IRT-based models. The Rasch model aims to look beyond a logistic function that relates the respondent’s underlying traits (or abilities) and item difficulty to the probability of endorsing an item [17]. Rasch models have been applied in many fields, such as health science, social psychology and education [15, 18].

Besides, the Rasch model has been increasingly applied to identify measurement issues not easily detected by CTT [15, 16, 18]. In the Rash model measures the only latent trait with a sufficient statistics for estimating the parameters of item difficulty and person ability [17]. Sufficient statistics allow the cumulative total raw scores acquired by counting the observed responses to be summated, which constructed item hierarchy structure how a person ability and item difficulty interact to regulate the probability of approving of an item along a construct continuum being measured. Furthermore, the Rasch model provides a proper method for converting the ordinal raw scores into interval measures (logit). Due to nonlinear transformation to interval measures, the Rasch model can allocate the person ability and item difficulty jointly onto the same interval scale [14] to allow for meaningful comparisons.

Although CTT-based methods have generally supported construct validity and internal consistency reliability of SGRQ, such methods cannot facilitate the evaluation of whether items are equivalent to different individuals. Lack of measurement equivalence may lead to incorrect estimates of effects in research and decision making [19]. One approach to understand scale equivalence in different groups or conditions is to use Item Response Theory (IRT)-based models [19, 20]. The situation where subjects from different groups, with the same level of the attribute, respond with different probabilities to endorse an items is defined as differential item functioning (DIF) [21]. The purpose of DIF is used to make sure whether the differences of item difficulty exist when measuring different group. Scales containing such DIF items have reduced validity for between-group comparisons because their scores are influenced by a variety of attributes other than those intended [19]. To date, most attention has been given to investigations of DIF associated with age [20, 22], sex [20, 22], culture [23] or, disease [24–26], but few studies have examined disease’s severity-related DIF.

The aim of this study attempted to apply the unique nature of Rasch model to rigorously evaluate the psychometric properties of the SGRQ questionnaire in COPD patients, both at the item and scale level in terms of dimensionality analysis and item fit evaluation. Specifically, item gaps along the construct continuum and the level of matching between the item difficulty and person ability (or traits) were examined for exploring possible scale modification. Finally, the analysis of differential item functioning (DIF) was performed based on different ages, and the disease’s severity of COPD patients.

## Methods

### Study sample

Patients diagnosed of COPD were recruited from the outpatient department of two teaching hospitals and two local hospitals in Central Taiwan. Patients with cognitive impairment or lung cancer were excluded. All consented patients were interviewed by a trained nurse and completed the SGRQ questionnaire. They also underwent a test of spirometry to collect patients’ FVC and FEV1 as a reference of severity classification of the GOLD [27]. The degree of spirometric abnormality generally reflects the severity of COPD. GOLD is abbreviated from Global Initiative for Chronic Obstructive Lung Disease, which announced by WHO in 2003 and became a global guideline for the diagnosis, management, and prevention of COPD (GOLD, 2007). This study was approved by the Institutional Review Board (IRB) of China Medical University Hospital (DMR94-IRB-179). Since very few female COPD patients were seen and enrolled in these hospitals, our data analysis focused only on those male patients.

### Instrument

The Taiwanese version of the SGRQ translated and validated by Wang et al. [28] was used in this study. It is a self-administered instrument for asthma and COPD that contains 50 items measuring three domains: Symptom (8 items), Activity (16 items) and Impact (26 items). In the Symptom domain, there are eight items about illness status such as cough, sputum production, and dyspnea (denoted as S_a group). Two items (Item S_a6 “How long did the worst attack of chest trouble last” and Item S_a8 “If you have a wheeze, is it worse in the morning”) that are not directly related to COPD were excluded from the psychometric analysis. In the Activity domain, there are 16 items separated into two groups: one with 7 items concerned with activities that cause breathlessness (denoted as A_c group) and the other with 9 items concerned with activities limited by breathlessness (denoted as A_g group). The Impact domain has 26 items that broadly assess the impact of the disease on the aspects of social, emotional functions and expectations for health (denoted as I_h - I_i group). The response options vary from 2 to 5-point ordinal scale depending on the type of question. Most items in the Activity and Impact domains use dichotomous (binary) response options (“true” or “false”) and most items in the Symptom domain use polytomous (multi-category) response options. Item scores in their respective domain were summed to arrive at a domain score and the Total score was as a percentage of overall impairment on quality of life, with higher scores indicating lower quality of life.

### Rasch model analysis

All Rasch model analysis were performed using the software of Winsteps (http://www.winsteps.com). Each of the three domains of SGRQ was tested separately. Since the SGRQ is composed of items with both dichotomous and polytomous response options, a mixed rating scale model and partial credit model by Winsteps were conducted. For items using the same response options such as those in the Activity and Impact domain, rating scale model was used [17]. For items with different sets of response options such as those in the Symptom domains, they were allocated into respective response option groups and analyzed by partial credit model [17, 29].

### Unidimensionality and local independence

Before Rasch model analysis was performed, it required the assessment of whether the SGRQ meets the test criteria of local independence and unidimensionality, which provides how well each item contributed to the single construct being measured [13]. To assess the property of local independence and unidimensionality of the Symptom domain, a confirmatory factor analysis (CFA) was conducted using the LISREL 8.51 software (Scientific Software International, Lincolnwood, IL). Unidimensionality was evaluated by the magnitude of factor loadings with a value > 0.3 indicative of importance, and three model fit indices – goodness-of-fit index (GFI), comparative fit index (CFI), Bentler-Bonett Normed Fit Index (NFI) - with an index > 0.9 indicative of good fit. The index standardized root mean square residual (SRMR) ≦0.08 was also used to evaluate the global model fit [30]. Cronbach’s coefficient Alpha (Kuder-Richardson formula 20, KR-20) was used to assess the unidimensionality of the Activity and Impact domains since only dichotomous item responses were used for the items in these two scales. Also, the test of dimensionality was undertaken by performing a principal component analysis (PCA) of the residuals derived from Rasch model analysis [24, 25]. If a scale is unidimensional, no residual associations within the first residual component should exist once the factor for which item associations exist is extracted. Local independence of item was considered that responding to one item should not influence the response to another item. It was verified with a correlation analysis of standardized residuals of the Rasch model analysis. High residuals correlations (|r| > 0.3) between any item pairs would violate local independence [22].

#### Item fit

After unidimensionality was established by either confirmatory factor analysis (CFA), Cronbach’s coefficient Alpha (KR-20) approach, or principal component analysis of the residuals, item fit was further examined to evaluate the item-level model fit. A good item fit referred to how well the observed data are close to the expected data. The items’ fit was provided by infit statistics, which is reported as mean square error derived from Rasch model analysis. The infit statistic gives relatively more weight to the performance of persons closer to the item value and can minimize the influence of outlying scores [14]. A value, either above 1.4 (misfitting) or below 0.6 (overfitting), indicates how well any set of empirical data met the compatibility with the model [31].

#### Reliability and separation index

The overall fit of each Domain of SGRQ to the Rasch model was determined by examining the person reliability and person separation index. The person reliability derived from Rasch model analysis was an indicative of internal consistency among all items within the same domain, which is analogous to the Cronbach’s coefficient Alpha. A value greater than 0.7 indicates good internal consistency or model fit [31]. The person separation index was used to indicate how efficiently a set of items within the same domain could distinguish the respondents’ traits and characteristics in the measure, with higher value indicates better separation. Values between 1.5 and 2.0 are considered to be acceptable and value higher than 3.0 suggest an excellent level of separation [31].

#### Item difficulty estimates

The Rasch model can provide an item and person estimate. The estimate of the item is called the item difficulty and referred to the location of the item on the logit scale. The estimate of the person is called the persons’ ability and informs about the ranking of each person on the same continuum. Since the estimates of the persons’ ability and the item difficulty are jointly placed on the same metric (called logit in the Rasch model), they can be compared, with a higher value indicating a more difficult item or a more able person. We used the item difficulty to evaluate the influence of COPD on patients in each domain. In the Symptom domain, an item with higher difficulty means that it is more difficult for that symptom not to occur or experience. In other word, an item with higher difficulty value is easier to occur in this case. In the Activity or Impact domain, an item with higher difficulty estimate means that it is more difficult for a person to achieve a level of activity or to perform a non-disturbance level of daily life. In order to minimize the gap and redundancy in item contents, the item difficulties should be evenly distributed to cover the entire test [32].

#### Targeting

Targeting is defined as the extent to which items are of appropriate difficulty for the sample [17]. A targeting index of zero (a perfect targeting) achieved would indicate the spectrum of item difficulties matching for the abilities of persons. A targeting index greater than 0 indicates that the subject tends to give ‘positive’ responses (e.g., ‘satisfied’), and that less than 0 indicates that the subject tends to give ‘negative’ responses (e.g., ‘dissatisfied’). The values of 0.5 to 1 or −0.5 to −1 were considered to be slight mis-targeting, and those greater than 1 or less than −1 to be substantially mis-targeting [31].

#### Range and gap

The range of item difficulty is the spread between the highest and lowest threshold values of all items within a domain. The coverage of the domain for person measures is defined as the percentage of people with a level of person measure within the highest and lowest thresholds. The value with at least 95 % coverage was considered to be a good fit [33]. A gap is defined as the difference between the two adjacent item difficulties, which are the average of thresholds of each item. When the value of item gap is ≧ 1 (logit), it implies that the items are not evenly distributed, or the items within domain are not sufficient [34].

#### Ceiling and floor effects

The ceiling effect of each domain is defined as the percentage of persons’ abilities greater than the highest threshold of item, and the floor effect as those less than the lowest threshold of item. A scale is free of the ceiling and floor effects when <15 % of persons’ abilities excel the most difficult threshold of item and <15 % of persons’ abilities are below the easiest threshold of item. Too “easy” or too “difficult” items would be recognized based on the person-item distribution. Higher percentages in these two extreme ends lead to lower reliability of discriminating respondents’ measures [33].

#### Item-person map

An item-person map locates along the same continuum where the estimates of sample respondent’s measure line up with the average difficulty of the items all on the same metric (the same line graphically). Many of the relationships between the estimates of person measure and item difficulty are shown graphically in two panels, where each individual person measure is represented by a symbol of “#” on the left panel and each item difficulty is indicated by the item number on the right panel. The value of the item-person map can easily look where the person measures are distributed in comparison with the item difficulties at a glance. Moreover, the gaps between items, the fit of person measure and item difficulty, and the floor or ceiling effect could be examined [17].

#### Differential item functioning (DIF) analysis

DIF analysis were investigated to generate group-specific estimates of the item difficulty driven from IRT method using Winsteps software. Comparing difference of the estimates of the item difficulty between subgroups was as a DIF contrast. Magnitude of DIF is defined that the DIF contrast greater than 0.5 logits is considered the existence of DIF [14, 35]. This study examined the age or severity of disease as a reference of DIF grouping. The research was not analyzed by the gender related DIF since our sample are all male. Age related DIF was analyzed using 75 years old as a divided reference due to the high prevalence of COPD among older population. Data from subgroups were computed separately and obtained the estimates of item difficulty for each group. Comparison of item difficulty estimates in each group was performed. Then the scatterings of item difficulty estimates of two subgroups were plotted based on age (age < 75, age ≧ 75) or severity of disease (stage012, stage34) as the corresponding x-axis and y-axis in Figs. 4 And 5. If two subgroups have the same difficulty of item, the estimate value of difficulty of the two subgroups will be centered near the 45° of diagonal line with a slope 1 in the scatter graph. The research used item difficulty difference greater than 0.5-logits as the criterion for detecting DIF. That is, 45° of diagonal solid line used as a base line and parallelly moving up or down 0.5-logits (2 dashed line). If the item DIF exists, the estimated value will fall over outside the range of the dashed line [14, 26].

## Results

### Sample characteristics

The age of the 240 male COPD patients ranged from 46 to 88 years, with a mean of 70.4 years. Most patients were married and had less than elementary school education, and over 25 % were current smokers. The predicted percent of FEV1 was 56.0 % and the average of FEV1/FVC ratio was 56.3 %. Patients were classified by the 2003 GOLD criteria into five groups based upon their severity of illness due to the collected time period. Most participants were in GOLD stage II or III. Detailed demographic and clinical characteristics of the study sample summarized in Table 1.

**Table 1:** Demographic and clinical characteristics of the study sample (N = 240 COPD patients)

Variables	Mean/n	SD/%
Sex: male	240	
Age: mean years (SD)	70.4	7.9
Lung function: mean (SD)		
FEV1 (% predicted)^a^	56.0	21.7
FEV1/FVC^b^ ratio (%)	56.3	12.5
Education: counts (%)		
Elementary school and below	158	66.11
Junior and senior high school	65	27.20
Junior college and above	16	6.69
Marital status: counts (%)		
Married & lives together	207	86.97
Other (unmarried, divorce, widower or widow)	31	13.03
Current smoking status: yes counts (%)	66	28.21
Disease severity: counts (%)		
Stage 0 & I: at risk or mild	39	16.25
Stage II: moderate	86	35.83
Stage III: severe	95	39.58
Stage IV: very severe	20	8.33

^a^FEV1 (% predicted) = forced expiratory volume in 1 s (% predicted)

^b^FVC = forced vital capacity

#### Unidimensionality and local independence

The results of the Symptom domain-specific CFA showed that the three fit indices were all <0.90 (GFI 0.853, CFI 0.662, and NFI 0.648) and the SRMR was 0.131. After one modification (justifying the relationship of the residual of S_a1 cough and S_a2 brought up sputum), the Symptom domain had GFI, NFI and CFI values were over 0.95 and SRMR value was less than 0.03. All standardized factor loadings for the Symptom domain items were above 0.4, with the exception of Item Sa_7 “How many good days have you had” being 0.26, supporting the assumption of unidimensionality in the Symptom domain. The Cronbach’s coefficient Alpha (KR-20) was 0.90 for the Activity domain and 0.88 for the Impact domain, indicating unidimensionality for these two domains. Unidimensionality was also affirmed in the PCA of residuals. The eigenvalue units of unexplained variance in 1st factor of the Symptom, Activity and Impact domains were 2.1, 2.3 and 2.7, respectively, indicating unidimensionality for all domains of SGRQ. After the confirmation of unidimensionality, local independence was examined by identifying correlations among the residuals of the items (residual |r| < 0.3). The range of all item residual correlations in the Symptoms Domain was −0.42 to 0.36, indicating a potential problematic dependence between items, especially in item S_a1 cough and S_a2 brought up sputum. High residual correlations in the Activity Domain was distributed over item A_g1, A_g6, and A_g8. High item dependence in the Impact Domain included item I_d6, I_e2, I_e8, I_h2, I_h3, I_h5.

#### Item fit

The infit statistics, item difficulties, their standard errors and separation indices for each domain are shown in Table 2. The ranges of the infit statistics of the Symptom, Activity and Impact domains were 0.85–1.46, 0.69–1.42 and 0.74–2.00, respectively. In the Symptom domain, item Sa_7 “How many good days (with little chest trouble) have you had” had an infit statistic of 1.46 and did not appear to fit the unidimensionality. In the Activity domain, item A_c7 “Playing sports or games” didn’t seem to fit the unidimensionality as its infit statistic was 1.42. In the Impact domain, item I_b1 “How would you describe your chest condition” didn’t fit the unidimensionality requirement as its infit statistic was 2.00. When these three items were excluded to refit each model separately, all remaining items in their respective domain of the SGRQ indicated good fit to the Rasch model.

**Table 2:** The model infit index, item difficulty parameters, and separation in the Symptom, Activity, and Impact domain of the SGRQ by the order of item difficulty

Domain/item	Infit index	Item difficulty (SE)	Reliability (separation index)
Symptoms domain			0.59 (1.21)
S_a5. Very bad unpleasant attacks of chest trouble^b^	0.85	−1.09 (0.06)	
S_a4. Attacks of wheezing	0.87	−0.18 (0.06)	
S_a3. Shortness of breath	0.90	0.09 (0.05)	
S_a7. How many good days (with little chest trouble)	1.46^a^	0.23 (0.06)	
S_a1. Coughed	0.87	0.47 (0.06)	
S_a2. Brought up phlegm (sputum)	1.08	0.47 (0.06)	
Activity domain			0.86 (2.50)
A_c1. Sitting or lying still	1.40	−6.42 (0.46)	
A_c2. Getting washed or dressed	0.93	−3.53 (0.24)	
A_g1. Take a long time to get washed or dressed	1.08	−3.14 (0.23)	
A_g2. Cannot take a bath or shower, or take a long time	0.90	−2.84 (0.22)	
A_c3. Walking around the home	0.86	−2.56 (0.21)	
A_c4. Walking outside on the level	0.82	−1.39 (0.20)	
A_g3. I walk more slowly than other people, or I stop for rests	1.09	−0.67 (0.19)	
A_g4. Jobs such as housework take a long time, or I have to stop for rests	0.81	0.04 (0.19)	
A_c5. Walking up a flight of stairs	0.92	0.38 (0.20)	
A_g5. If walk up one flight of stairs, I have to go slowly or stop	0.78	0.61 (0.20)	
A_c7. Playing sports or games	1.42^a^	2.54 (0.24)	
A_g7. Walk up hills, carry things up stairs or play golf	0.80	2.71 (0.24)	
A_c6. Walking up hills	1.35	2.77 (0.24)	
A_g6. If hurry or walk fast, I have to stop or slow down	0.86	2.96 (0.25)	
A_g8. Carry heavy loads, jog or walk at 5 miles per hour or swim	0.69	4.07 (0.30)	
A_g9. Very heavy manual work, run, cycle, play competitive sports	0.86	4.46 (0.32)	
Impact domain			0.81 (2.08)
I_h5. Move far from my bed or chair	0.94	−1.87 (0.26)	
I_f2. I get embarrassed using my medication in public	1.13	−1.30 (0.22)	
I_f1. My medication does not help me very much	1.25	−1.30 (0.22)	
I_e5. I do not expect my chest to get any better	0.90	−0.91 (0.20)	
I_d3. I get breathless when I talk	0.76	−0.72 (0.19)	
I_d4. I get breathless when I bend over	0.84	−0.62 (0.19)	
I_h3. Go out of the house to do the shopping	0.88	−0.54 (0.18)	
I_f3. I have unpleasant side effects from my medication	1.17	−0.54 (0.18)	
I_f4. My medication interferes with my life a lot	1.07	−0.51 (0.18)	
I_h4 Do housework	0.87	−0.50 (0.18)	
I_d1. My cough hurts	0.87	−0.45 (0.18)	
I_e2. My chest trouble is a nuisance to my family, friends or neighbors	0.99	−0.35 (0.18)	
I_b2. Chest trouble affect my work	1.21	−0.20 (0.12)	
I_e3. I get afraid or panic when I cannot get my breath	0.96	−0.17 (0.17)	
I_i. Best describes how your chest affects you^b^	0.98	−0.13 (0.10)	
I_e4. I feel that I am not in control of my chest problem	0.89	−0.11 (0.17)	
I_h2. Go out for entertainment or recreation	0.80	0.15 (0.16)	
I_e1. My cough or breathing is embarrassing in public	1.16	0.24 (0.16)	
I_d2. My cough makes me tired	0.94	0.88 (0.15)	
I_d5. My cough or breathing disturbs my sleep	1.13	0.93 (0.15)	
I_h1. Play sports or games	0.88	1.11 (0.15)	
I_b1. How would you describe your chest condition	2.00^a^	1.15 (0.08)	
I_d6. I get exhausted easily	0.90	1.19 (0.15)	
I_e6. I have become frail or an invalid because of my chest	0.74	1.21 (0.15)	
I_e8. Everything seems too much of an effort	0.76	1.62 (0.15)	
I_e7. Exercise is not safe for me	0.89	1.75 (0.16)	

Estimates were obtained from a mixed rating scale and partial credit model by Winsteps

^a^Item misfit (infit index > 1.4) ^b^Reversed item

#### Reliability and separation index

The person reliability for each of the three SGRQ domains was acceptable, with the reliability coefficient ranging from 0.81 (Impact domain) to 0.86 (Activity domain) except the Symptom domain (0.59). The person separation indices for the Symptom, Activity, and Impact domains were 1.21, 2.50, and 2.08, respectively. Most of these domains had acceptable separation properties except the Symptom domain (Table 2).

#### Item difficulty estimates

The results showed that item difficulties of each domain, especially those in the Activity domain were hierarchically ordered along the logit metric (see Table 2). In the Symptom domain, the occurrence of symptoms increased with the level of item’s difficulty. That is, the symptoms of highest occurrence across COPD population were cough as well as spitting and the symptom of least occurrence was S_a5 “Very bad unpleasant attacks of chest trouble”. In the Activity domain, the item difficulty was listed from the least exertional activities (e.g., sitting or lying) to the most exertional activities (e.g., running, playing competitive sports). In the Impact domain, the most difficult items (e.g., items with difficulty greater than 1) were clustered around the impact events induced by activities with exertion.

#### Targeting

The mean values of targeting indices varied across the three domains. The value of 0.08 was near 0 for the Symptom domain, indicating a good match. The value of −0.14 indicates slight difficulty in the Activity domain. In contrast, a value of 1.36 indicated that the Impact domain was easier for patients. The results indicated that this study population had higher tendency selecting more ‘positive’ response options in each domain, except the Activity domain.

#### Ranges and gaps

The ranges of the thresholds in each domain were −3.03 to 1.27 for the Symptom domain, −6.42 to 4.46 for the Activity domain, and −1.87 to 2.39 for the Impact domain (Table 3). The distribution of 95 % person measure ranges were −1.65 ~ to 2.51, −7.47 ~ to 6.48, −1.59 ~ to 5.11 for the Symptom, Activity, and Impact domain, respectively. Thus, these item thresholds ranges cover 91.67 %, 88.75 %, 72.92 % of respondents for respective domain, indicating that the SGRQ provided a satisfactory estimation for most patients in this study. No obvious gaps in the Symptom and Impact domains were observed (see Table 2). However, there were obvious gaps in the Activity domain between A_c1 and A_c2, between A_c3 and A_c4, between A_g5 and A_c7, between A_g6 and A_g8.

**Table 3:** Distribution of the item thresholds and person measures by the SGRQ domain

	Symptoms domain	Activity domain	Impact domain
Range of item threshold	−3.03 – 1.27	−6.42 – 4.46	−1.87 – 2.39
Range of person measure	−4.16 – 3.75	−7.52 – 6.48	−3.72 – 5.11
95 % person measure limits^a^	−1.65 – 2.51	−7.47 – 6.48	−1.59 – 5.11
Coverage (%)^b^	91.67	88.75	72.92
Floor effect (%)	0.83	2.50	0.83
Ceiling effect (%)	7.50	8.75	26.25

^a^The 2.5th percentile to 97.5th percentile of person ability

^b^The percentage of person measures that fall between the lowest and highest item thresholds

#### Floor and ceiling effects

The results of floor and ceiling effects are summarized in Table 3. Most results of floor and ceiling effects were less than 10 %, while the ceiling effect in the Impact domain was three times higher than that in the other two domains.

#### Item-person map

**Fig. 1:**
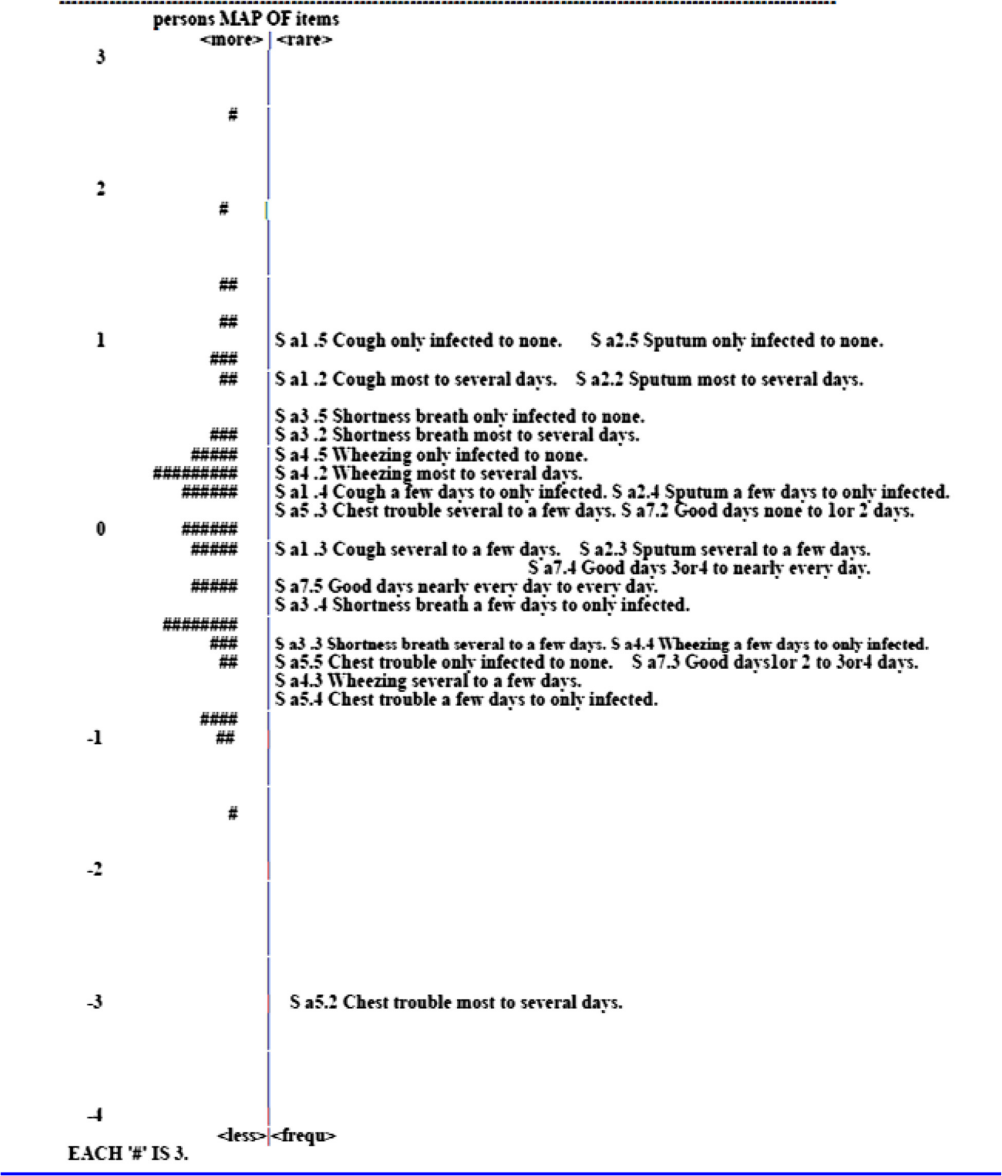
Item-person map on the logit scale for the Symptom domain of the SGRQ

**Fig. 2:**
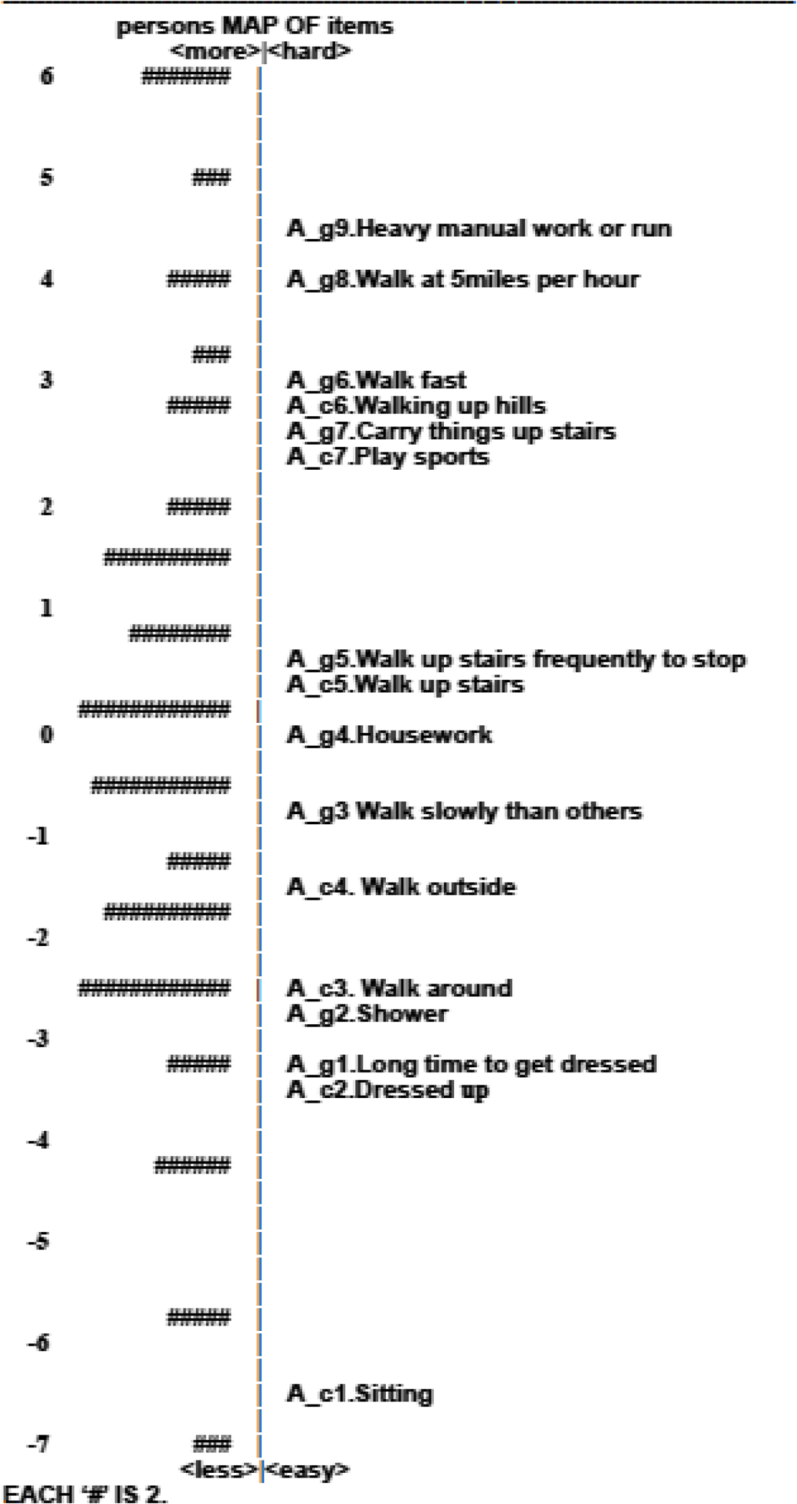
Item-person map on the logit scale for the Activity domain of the SGRQ

**Fig. 3:**
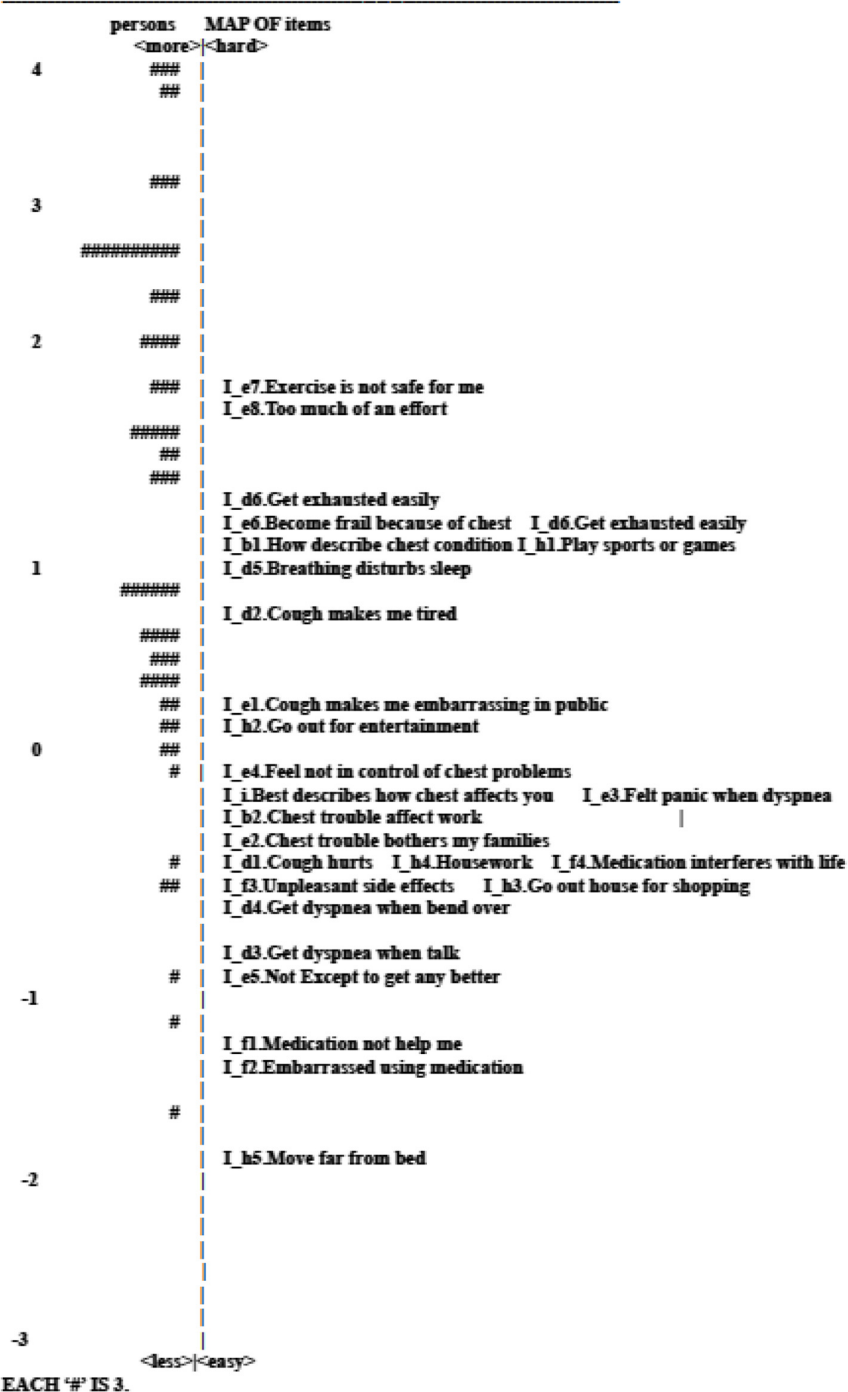
Item-person map on the logit scale for the Impact domain of the SGRQ

Figures 1, 2 and 3 are the item-person maps for the Symptom, Activity and Impact domains depicting the estimates of person measure (the left panel) and sets of threshold parameter estimates of item difficulty (the right panel) on the same “logit” scale. On the right panel of the map of the Symptom domain, the digit after the decimal point in the name of each item denoted a certain threshold. For example, S_a1.2 indicates the first threshold in which the respondents had equal 50 % of chance of choosing either first or second option in the S_a1 item. As modeled, there were 4 thresholds for an item with 5 response options and they were symbolized as S_a1.2, S_a1.3, S_a1.4 and S_a1.5. For the other two domains, no digit after the decimal was shown because all items were with binary response resulting only one threshold. In the Symptom domain, the estimates of person ability and item difficulty were mostly scattered between −1 and 1 (Fig. 1). The structure of thresholds was disordered in the Symptom domain. In the Activity domain, a hierarchical structure was shown as expected, whereas there were quite a few gaps shown, especially the gap between A_c1 and A_c2 as well as A_g5 and A_c7 (Fig. 2). In addition, there were no suitable items to discriminate persons whose ability fell into the extreme ability level (ability level between 5 ~ 6 or −4 ~ −6). In the Impact domain, a reasonably ordered structure was represented as expected, but the range of item difficulties could not cover that of person measures especially at the high end of respondent’s measures (Fig. 3).

### Differential item functioning (DIF) analysis

1. The age related DIF

**Fig. 4:**
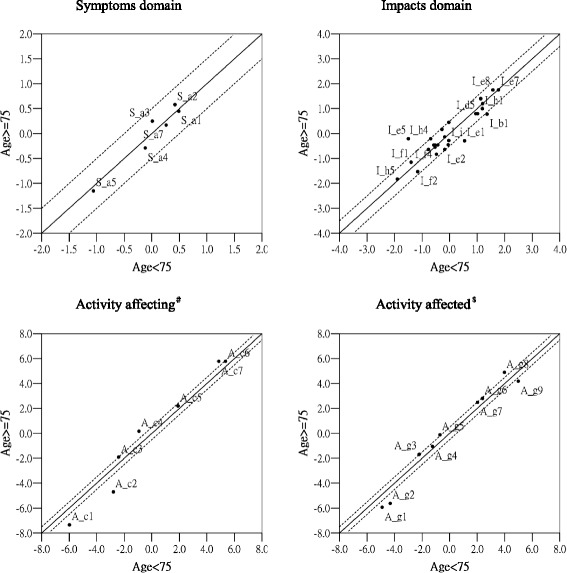
Differential item functioning plots the scatterings of item difficulty (in logits) for each domain of the SGRQ items by age group comparison. ^#^Questions about what activities usually make you feel breathless. ^$^Questions about how activities may be affected by your breathing

Figure 4 is a scatter showing differential item functioning plots for each domain of the SGRQ item by the age comparison. In the Symptom domain, there is no DIF. In the Activity domain of the set of 「What activities usually make you feel breathless」, 4 (57 %) of 7 questions have the existence of DIF, while there is 6 (67 %) of 9 questions in the set of how does the problem of respiratory affect your activity. In the Impact domain, there is DIF in the 3 (12 %) of 26 questions.

(2) The severity related DIF

**Fig. 5:**
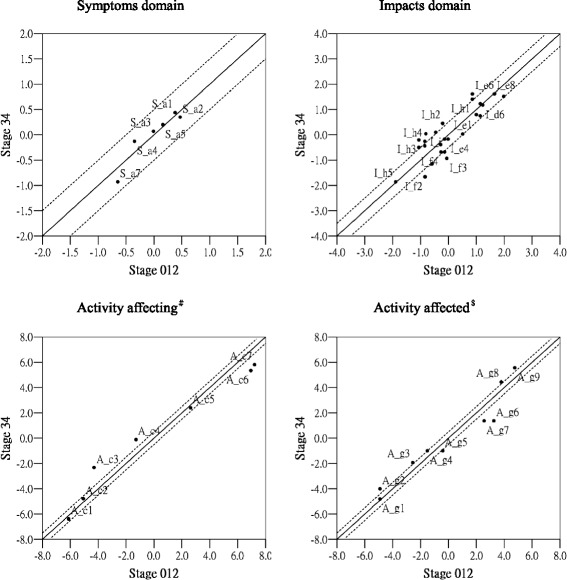
Differential item functioning plots the scatterings of item difficulty (in logits) for each domain of the SGRQ items by disease severity group comparison. ^#^Questions about what activities usually make you feel breathless. ^$^Questions about how activities may be affected by your breathing

Figure 5 is a scatter showing differential item functioning plots for each domain of the SGRQ items by the disease severity comparison. In the Symptom domain, there is no DIF. In the Activity domain of 「what activities usually make you feel breathless」, 4 (57 %) of 7 questions have the existence of DIF, while there is 8 (89 %) of 9 questions in 「how does the problem of respiratory affect your activity」. In the Impact domain, there is DIF in the 13 (50 %) of 26 questions.

## Discussion

One advantage of Rasch model analysis is to allocate the person abilities and item difficulties jointly onto the same interval scale, which can serve as a guidance to revise or refine the questionnaire or test items. This study applied the Rasch model to rigorously examine the psychometric properties of the SGRQ in patients with COPD at both domain and item levels. The results showed that most items within their respective domain had a goodness-of-fit for unidimensionality. These findings were similar to those reported by Meguro [36]. Moreover, each domain of the SGRQ reported good person reliability and separation, except the Symptom domain, which is similar to the result of CTT analysis in the previous study [37] and by IRT [36]. As the sample of this study had a wide range of disease severity (including ‘at risk’ to ‘severe’ group), the characteristics of the patient group had a greater variety of illness symptoms, leading to low person reliability and separation. While beyond our imagination, most items in the Symptom domain exhibited disordered thresholds, which were similar to those in Meguro et al.’s study [36]. One possible explanation for this phenomenon is that symptoms varied considerably among patients due to the nature of COPD [37]. Furthermore, the wording of response options might lead to disordered thresholds [18, 36]. They have suggested that the scaling property of the ordered response options for the Symptom domain could be improved by combining two or more ambiguous categories [18, 36]. We have revised our scaling based on their suggestion for modification; however, the disordered thresholds of the Symptom domain were not completely improved. We collapsed some of the response options from 5 response choices to 3 or 4, as described below, and this solved the phenomenon of disordered thresholds in our data. For the items S_a1 to S_a4, we combined “a few days a month” and”several days a week” into one category (denoted as “several days”) to form 4 response choices, which were “not at all”,“only with chest infection”, “several days” and “most days”. For item S_a5 “how many severe or very unpleasant attacks have you had”, the 5 response choices were combined into 3 response choices: “no attacks”, “1 or 2 attacks” and “3 or more attacks”. And for item S_a7 “how many good days have you had”, the 5 response choices were combined into 3 response choices: “no days”, “some or a few days” and “every day”. The results of thresholds in the Symptom domain after revision were shown in the Table and Figure (see Appendix).

When the scale had a clear gradient of difficulty level across a set of items, Rasch model, as compared with the CTT, could exhibit its psychometric properties, such as item hierarchy, item redundancy and gaps of the scale more structurally [38]. The results showed that the item difficulty in the Activity domain of the SGRQ gave a remarkably clear gradient activity from low exertion (e.g., Sitting or lying) to high exertion (e.g., running). In the Activity domain, there are two groups of items: “what activities make you feel breathless (group of A_c)” and “how activities may be affected by your breathing (group of A_g)”. An analysis of the estimates of item difficulty in these two sets showed that some items may be redundant (Table 2 and Fig. 2). For example, “A_c2 Getting washed or dressed” was similar to “A_g1 Take a long time to get washed or dressed”, and “A_c6 Walking up hills” was similar to “A_g7 Walk up hills, carry things up stairs or play golf”. Consequently, some items could be considered as possible candidates for item removal in order to improve tool efficiency. Moreover, there were apparent gaps between some items (Table 2 and Fig. 2), especially between items A_cl and A_c2, as well as items A_g5 and A_c7. These gaps indicated that some new items may be necessary to fill those gaps and cover the continuum in order to able to better differentiate the respondents’ abilities.

The Rasch model places the person measures and item difficulties on the same metric, allowing the identification of the level of matching between the item difficulty and the person ability. Our results showed that the targeting and the ceiling effect were high and the percentage of the coverage of the scale was low in the Impact domain compared to other domains. This showed that the items of the Impact domain were too simple for respondents with high ability to discriminate (such as at stage 0 & I). In the Impact domain, most items calibrated in the difficult end were related to the impact of daily life, which was caused by the activity with more effort. However, for most COPD patients in the early stages, they are generally not frail, which caused the high ceiling effect in our results. When the revision of the SGRQ is considered, it is imperative to increase the difficulty of some items and to add more items related to psychosocial adjustment, such as sense of control, in the Impact domain in order to reflect the psychosocial impact of the beginning of the illness. This would better discriminate the impact of COPD at different stages.

Establishing measurement equivalence is important because lack of measurement equivalence may lead to incorrect estimates of effects in research [19]. Examination of DIF was to identify whether the item parameters will be invariant across the different subgroups. The results of this study showed that many items of SGRQ presented the age or severity related DIF, indicating somewhat unstable across different characteristics of group. In terms of the age related DIF, the effect of age on the Symptom and Impact domain of SGRQ was not much, but there was many DIF in the Activity domain, which implied age could be affected by underlying physical function to cause difference in a certain degree. Likewise, many items had the severity related DIF in the Activity and Impact domain of SGRQ, indicating the different stage of disease in COPD patients will bring the different results of the disease’s impact.

In spite of higher ratio of DIF in the Activity domain of SGRQ, the conformation of DIF exists most in the easiest and hardest end. Further investigation would find the similiar item hierarchy across different subgroups. The phenomena that more DIF exists in the Activity domain of SGRQ may be caused by an obvious difficulty gradient of underlying physical function. Furthermore, the analysis of DIF will be affected by response option. Multiple response option can have better ability to differentiate the results. However, the items in the Activity domain of SGRQ is dichotomous option response, so the items were easily prone to present DIF. Compared to the age related DIF, the severity related DIF exists more. This phenomenon was justified that the SGRQ is developed by specific disease and this kind of design may facilitate the DIF to become more apparent. Although the disease’s severity and age rendered some DIF, the existence of DIF within the health assessment can be considered as a sensitive measurement to differentiate the impact of quality of life that affected by disease’s severity or age across subgroups [26, 39]. Although the result had a high proportion of DIF, it doesn’t mean that questionnaire is not applicable, which rather represent that these items may be suitable for developing the computer adaptive test. Questionnaire developer can use a few items to obtain almost the same accuracy as the result get from the original questionnaire with more items.

There are a few limitations in this study. First, this study was a cross-sectional, so responsiveness to changes at different time points could not be assessed. Second, the study population included only male COPD outpatients and predominantly in GOLD stages II and III. In Taiwan, smoking is prevalent (approximately 54 %, including ex-smokers) among males over 50 years, compared with only about 4 % in females in the same age group in 2001 [40]. There are relatively few female patients with COPD compared with males in the clinical setting. Thus, we focused our analysis on male COPD patients. Consequently, the results of this study may not be applicable to female, hospitalized, or more severe patients with COPD. Furthermore, results were obtained only those patients whose conditions were stable enough to complete the questionnaires and could tolerate the interview and, thus, the final sample might have exluded patients with severe conditions. The domain scores might, therefore, have been better they were included in this study.

## Conclusions

In conclusion, our study highlights that a robust statistical technique in terms of Rasch model analysis was used to rigorously examine the psychometric properties of the SGRQ. The Rasch model facilitates disclosure of measurement problems that may not be easily detected by traditional analyses. Rasch model allows estimates of item difficulty and person ability spread along postulated latent traits and in ordered continuum that enables the examination of the hierarchical structure, targeting, and DIF of SGRQ. Hence, the results of Rasch model analysis provided a comprehensive basis for researchers to revise or develop the questionnaire, and highlighted aspects for improvement, such as gap, and duplicate items. There was some DIF existence in the Activity and Impact domain of SGRQ because SGRQ was a disease specific questionnaire and dichotomous response options, which may make more sensitive to detect disease’s impact. DIF assessment of measures remains an important component of efforts to achieve measurement equivalence in an increasingly heterogeneous society and may be workable to be used to develop the computer adaptive test.

## Figures and Tables

**Fig. 6:**
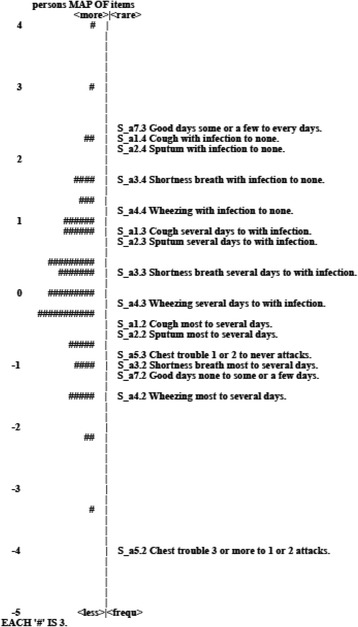
Revised item-person map on the logit scale for the Symptom domain of the SGRQ

## Appendix

**Table 4:** Revised thresholds and response categories for the Symptom domain of the SGRQ

	Thresholds between response categories (logits)		
Item	“Most days”/“several days”	“Several days”/“only with infection”	“Only with infection”/“not at all”
S_a1	0.14	0.87	1.53
S_a2	0.13	0.86	1.51
S_a3	–0.43	0.30	0.96
S_a4	–0.82	–0.09	0.57
Item	3 or more attacks/1 or 2 attacks	1 or 2 attacks/no attacks	
S_a5	–3.88	–0.93	
Item	no days/some or a few days	some or a few days/every day	
S_a7	–1.07	2.19	

## Abbreviations

COPD: Chronic obstructive pulmonary disease
PRO: Patient-report outcomes
HRQOL: Health-related quality of life
FDA: Food and Drug Administration
SGRQ: St. George’s Respiratory Questionnaire
CTT: Classical Test Theory
IRT: Item Response Theory
RSM: Rating scale model
CFA: Confirmatory factor analysis
GFI: Goodness-of-fit index
CFI: Comparative fit index
NFI: Normed Fit Index
SRMR: Standardized root mean square residual
KR-20: Kuder-Richardson formula 20
PCA: Principal component analysis
